# Synthesis and *In Vitro* Evaluation of *N*-(Bromobut-3-en-2-yl)-7-methoxy-1,2,3,4-tetrahydroacridin-9-amine as a Cholinesterase Inhibitor with Regard to Alzheimer's Disease Treatment

**DOI:** 10.3390/molecules15128804

**Published:** 2010-12-02

**Authors:** Jan Korabecny, Kamil Musilek, Ondrej Holas, Eugenie Nepovimova, Daniel Jun, Filip Zemek, Veronika Opletalova, Jiri Patocka, Vlastimil Dohnal, Florian Nachon, Jana Hroudova, Zdenek Fisar, Kamil Kuca

**Affiliations:** 1 Department of Pharmaceutical Chemistry and Drug Control, Faculty of Pharmacy in Hradec Kralove, Charles University in Prague, Heyrovskeho 1203, 500 05 Hradec Kralove, Czech Republic; E-Mail: korabecny.jan@gmail.com (J.K.); 2 Department of Toxicology, Faculty of Military Health Sciences, Trebesska 1575, 500 01 Hradec Kralove, Czech Republic; 3 Center of Advanced Studies, Faculty of Military Health Sciences, Trebesska 1575, 500 01 Hradec Kralove, Czech Republic; E-Mail: kucakam@pmfhk.cz (K.K.); 4 Department of Radiology and Toxicology, Faculty of Health and Social Studies, University of South Bohemia, Ceske Budejovice, Czech Republic; E-Mail: prof.patocka@gmail.com (J.P.); 5 Department of Chemistry, Faculty of Sciences, J.E. Purkinje University, Usti nad Labem, Czech Republic; 6 Department of Toxicology, Research Center of Military Health Service (CRSSA), 38702 La Tronche Cedex, France; E-Mail: florian@nachon.net (F.N.); 7 Department of Psychiatry, First faculty of Medicine, Charles University in Prague and General University Hospital in Prague, Prague, Czech Republic

**Keywords:** tacrine, acetylcholinesterase, butyrylcholinesterase, 7-MEOTA, inhibition, Alzheimer disease

## Abstract

A new tacrine based cholinesterase inhibitor, *N*-(bromobut-3-en-2-yl)-7-methoxy-1,2,3,4-tetrahydroacridin-9-amine (**1**), was designed and synthesized to interact with specific regions of human acetylcholinesterase and human butyrylcholinesterase. Its inhibitory ability towards cholinesterases was determined and compared to tacrine (THA) and 9-amino-7-methoxy-1,2,3,4-tetrahydroacridine (7-MEOTA). The assessment of IC_50_ values revealed **1** as a weak inhibitor of both tested enzymes.

## 1. Introduction

Alzheimer's disease (AD) is an age-related progressive neurodegenerative disorder that represents an intensive medical need [1]. It is the most common form of dementia, affecting about 5% of adults over 65 years. Overall, AD is the fifth leading cause of death for US citizens aged 65 and older. The most common symptom is known as progressive difficulties in remembering new information. No treatment is currently available to slow or stop the deterioration of the brain cells in AD [1,2].

The destruction of cholinergic neurons in the basal forebrain and reduction of choline acetyltransferase (EC 2.3.1.6; ChAT) and acetylcholinesterase (EC 3.1.1.7; AChE) activity in brain tissues in AD patients were first reported in 1976 and 1977 [3,4]. Thus, recent pharmacological research suggests that dysfunctions of specific cholinergic mechanisms may be partially responsible for the decline in memory observed in older age. AD treatment has focused on reducing cognitive decline with cholinesterase inhibitors (ChIs), the treatment of first choice currently available for this purpose.

**Figure 1:**
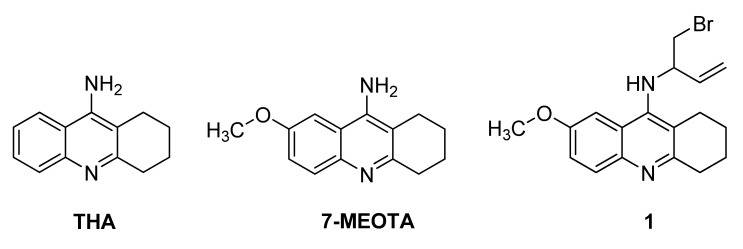
Structures of THA, 7-MEOTA and analogue **1**.

Tacrine (9-amino-1,2,3,4-tetrahydroacridine; THA; Figure 1) was an effective drug in the treatment of AD [5]. It was approved for clinical use by the U.S. FDA in 1993. However, it exhibited hepatotoxicity via elevation of serum alanine aminotransferase levels and thus showed limited clinical application and it was withdrawn from the pharmaceutical market shortly after its approval. In contrast, 7-MEOTA (9-amino-7-methoxy-1,2,3,4-tetrahydroacridine; Figure 1) was found to be a potent, centrally active ChI free of the serious side effects related to tacrine [6,7,8,9]. In single administration studies, 7-MEOTA was well-tolerated and thus further research efforts are currently aimed at improving its pharmacological profile [10]. The synthesis and biological evaluation of some 7-MEOTA derivatives as novel anti-AD agents was already reported [11,12].

In this study the synthesis, biological evaluation and molecular docking of the potential anti-AD agent *N*-(bromobut-3-en-2-yl)-7-methoxy-1,2,3,4-tetrahydroacridin-9-amine (**1**, Figure 1). 

## 2. Results and Discussion

**Scheme 1:**
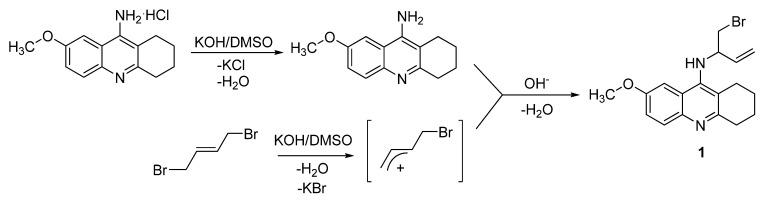
Proposed mechanism of 7-MEOTA reaction with (*E*)-1,4-dibromo-but-2-ene.

Compound **1** has been synthesized by a nucleophilic substitution reaction. Firstly, 9-amino-7-methoxy-1,2,3,4-tetrahydroacridine (7-MEOTA) was deprotonated using potassium hydroxide in DMSO at room temperature and the aromatic amine of 7-MEOTA was thus prepared. The planned reaction presumed nucleophilic substitution of 7-MEOTA with (*E*)-1,4-dibromo-but-2-ene and formation of 7-MEOTA dimer – 9,9`-((*E*)-but-2-ene-1,4-diyldiimino)-di-(7-methoxy-1,2,3,4-tetra-hydroacridine) – but this dimer was not obtained and a different product was produced by a different mechanism that involved in the first step a β-elimination reaction (Scheme 1) with a bimolecular transition state. The β-proton of (*E*)-1,4-dibromo-but-2-ene was removed via leaving group assistance and a carbocation was produced. This activated carbocation was coupled with 7-MEOTA base via nucleophilic substitution and *N*-(bromobut-3-en-2-yl)-7-methoxy-1,2,3,4-tetrahydroacridin-9-amine (**1**) was thus formed (Scheme 1, compound **1**) [13], although in very low yield (2%). This mechanism has not been previously described.

**Figure 2:**
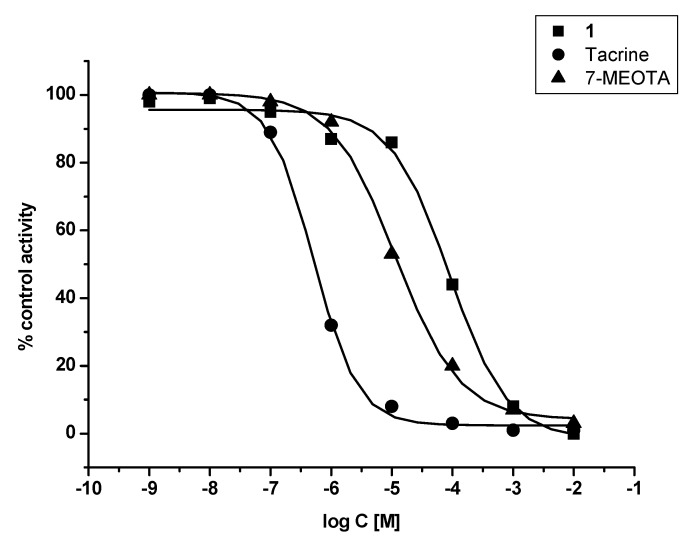
Enzyme activity plot of **1**, THA and 7-MEOTA for hAChE.

**Figure 3:**
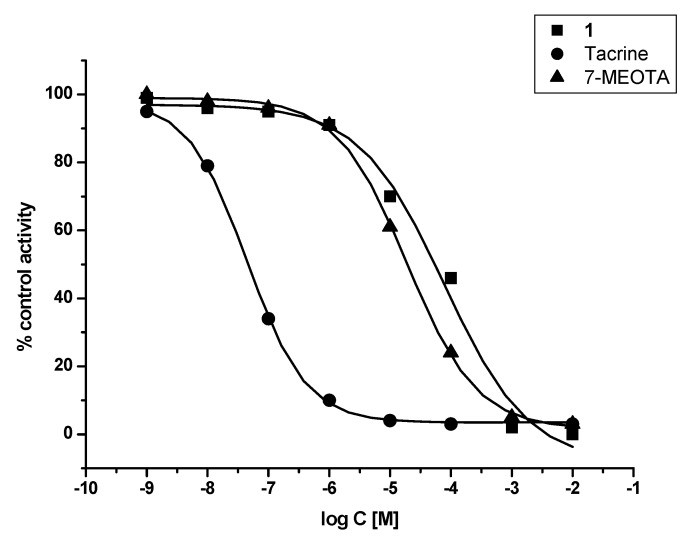
Enzyme activity plot of **1**, THA and 7-MEOTA for hBChE.

*In vitro* assessment showed that **1** was weak a ChI compared to THA and 7-MEOTA for both tested enzymes – human recombinant AChE (hAChE) and human plasmatic BChE (hBChE). Additionally, **1** was found to be a two orders of magnitude worse inhibitor of hAChE when compared with THA and a three orders of magnitude weaker inhibitor of hBChE in comparison with THA. Similar results were observed comparing **1** to its parent compound (7-MEOTA), where 7-MEOTA showed better inhibitory ability towards hAChE and hBChE (Figure 2 and Figure 3). The selectivity index (SI) for hAChE was calculated (Table 1). Comparing SI, **1** was more than fifteen fold more selective towards hAChE than THA, but it didn’t exceed 7-MEOTAwhich was a slightly more selective inhibitor of hAChE.

**Figure 4:**
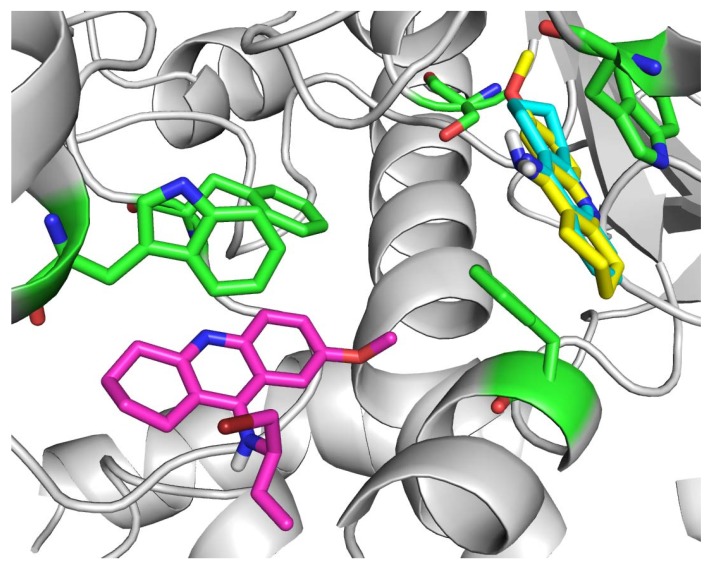
Molecular docking results for THA, 7-MEOTA and compound 1.

Molecular docking was performed in order to rationalize the *in vitro* findings. The tAChE crystal structure with THA (1acj) was chosen as a relevant enzyme source due to the similar molecular structures of THA, 7-MEOTA and compound **1** [14]. The THA molecule (in blue) remained the same as in the acquired crystal structure and is visualized together with the top-scored docking poses of 7-MEOTA molecule (in yellow; Figure 4). Both compounds displayed binding to the cation-π site of tAChE with very similar spatial orientations. The crystal structure of THA is stacked between Trp84 (3.6 Å) and Phe330 (3.8 Å) and it is further interacting with Tyr334 (3.7 Å), Trp432 (3.5 Å), His440 (4.3 Å) and Tyr 442 (3.8 Å). Additionally, the 9-amino moiety is attached by hydrogen bonds to two water molecules (2.1 and 2.2 Å).

Similarly, the top-scored docking pose of 7-MEOTA (-8.24 kcal/mol) showed apparent π-π interactions with Trp84 (3.6 Å) and Phe330 (3.8 Å). However, it resulted 180 degrees rotated from THA with same position of the 9-amino moiety, whereas the hydrogen bonds with two water molecules (both 2.2 Å) remained similar and its 7-methoxy moiety was attached by hydrogen bond to Gly118 (3.3 Å). This rotation of 7-MEOTA may explain its lower inhibitory ability towards AChE (15 µM) compared to THA (0.5 µM), where the aromatic ring with 7-methoxy moiety remained a bit distant from other important AChE aromatic residues (Tyr334, Trp432, Tyr442).

The top-scored docking pose of compound **1** (-5.60 kcal/mol) displayed a different type of binding, interacting with tAChE residues of the peripheral active site (PAS). Importantly, it was attached to Trp279 (3.6 Å) and Phe290 (3.7 Å) by π-π interactions. Additionally, the aromatic nitrogen and secondary amino group showed interactions with two water molecules (2.0 and 2.3 Å). Its binding at the rim of the AChE gorge may well justify its low *in vitro* inhibitory ability (89 µM), where it was not able to close the AChE gorge and prevent to decomposition of the substrate (acetylthiocholine).

## 3. Experimental 

### 3.1. Chemistry

7-MEOTA was prepared at our department according to the method described earlier [15]. All reagents were obtained from commercial sources in reagent grade quality. All experiments were carried out under nitrogen atmosphere. Thin layer chromatography (TLC) was performed on aluminium sheets precoated with silica gel 60 F_254_ (Merck). Column chromatography was performed at normal pressure on silica gel 100 (particle size 0.063-0.200 mm, 70-230 mesh ASTM, Fluka). Elemental analyses were obtained on a Perkin-Elmer CHN Analyser 2400 Serie II apparatus. Mass spectra were recorded using a combination of high performance liquid chromatography and mass spectrometry. The HP1100 HPLC system was obtained from Agilent Technologies (Waldbronn, Germany). It consisted of a G1322A vacuum degasser, G1311A quaternary pump, G1313A autosampler and a MSD1456 VL quadrupole mass spectrometer equipped with an electrospray ionization source. Nitrogen for the mass spectrometer was supplied by a Whatman 75-720 nitrogen generator. Data were collected in positive ion mode with an ESI probe voltage of 4,000 V. The pressure of nebulizer gas was set up to 35 psig. Drying gas temperature was operated at 335 °C and flow at 13 L/min. ^1^H-NMR and ^13^C-NMR spectra were recorded with a Varian Mercury VX BB 300 spectrometer operating at 300 and 75 MHz, respectively, using tetramethylsilane (TMS) as reference. Chemical shifts are reported in parts per milion (ppm, δ) relative to TMS. Melting points were measured on a micro heating stage PHMK 05 (VEB Kombinant Nagema, Radebeul, Germany) and are uncorrected.

### 3.2. *In vitro* evaluation

A Sunrise multichannel spectrophotometer (Tecan, Salzburg, Austria) was used for all cholinesterase activity measurements. A previously optimized Ellman procedure was slightly modified in order to estimate anticholinergic properties [16]. 96-well photometric microplates made from polystyrene (Nunc, Rockilde, Denmark) were used for measuring purposes. Human recombinant AChE or human plasmatic BChE (Aldrich; commercially purified by affinity chromatography) were suspended into phosphate buffer (pH 7.4) up to final activity 0.002 U/µL. Cholinesterase (5 µL), freshly mixed solution of 0.4 mg/mL 5,5´-dithio-bis(2-nitrobenzoic) acid (40 µL), 1 mM acetylthiocholine chloride in phosphate buffer (20 µL) and appropriate concentration of inhibitor (1 mM-0.1 nM; 5 µL) were injected per well. Absorbance was measured at 412 nm after 5 minutes incubation using automatic shaking of the microplate. The obtained data were used to compute percentage of inhibition (I; Equation (1)):

(1) $$I=1-\frac{\Delta A_{i}}{\Delta A_{0}}\;[\%]$$

where ΔA_i_ indicates absorbance change provided by cholinesterase exposed to AChE inhibitors and ΔA_0_ indicates absorbance change caused by intact cholinesterase (phosphate buffer was applied instead of AChE inhibitor). IC_50_ values were calculated using Origin 6.1 (Northampton, MA, USA). Percentage of inhibition for the given anticholinergic compound was overlaid by proper curve chosen according to optimal correlation coefficient. IC_50_ as well as upper limit of inhibition (maximal inhibition provided by given compound) was computed.

### 3.3. Synthesis of N-(bromobut-3-en-2-yl)-7-methoxy-1,2,3,4-tetrahydroacridin-9-amine *(**1**)*

Powdered potassium hydroxide (636 mg, 11.3 mmol) was added to a solution of 7-MEOTA (1,000 mg, 3.8 mmol) and DMSO (20 mL). The mixture was vigorously stirred at room temperature for 2 hours. Then (*E*)*-*1,4-dibromo-but-2-ene (808 mg; 3.8 mmol) was added and the stirring was continued for further 48 hours at room temperature. The end of reaction was determined on a triethylamine-saturated TLC plate, mobile phase hexane/ethyl-acetate (1:1). Water (100 mL) was poured into the reaction mixture and the water phase was extracted with ethyl acetate (4 × 60 mL). The combined organic extracts were dried over anhydrous sodium sulfate, filtered and evaporated under reduced pressure to give an oily residue that was purified by column chromatography on triethylamine-saturated silica gel. A 1:1 hexane/ethyl-acetate mobile phase was used for purification. The pure product was isolated as brown crystals [11,12,17]. Yield: 20 mg (2%); m.p. 100.8-103.9 °C; ^1^H-NMR (chloroform-d_3_): 7.81 (d, *J* = 9.08 Hz, 1H, -O-C-CH-CH-), 7.34 (d, *J* = 2.64 Hz, 1H, -O-C-CH-C-), 7.23 (dd, *J*_1_ = 2.64 Hz, *J*_2_ = 9.08 Hz, -O-C-CH-CH-), 5.97-5.82 (m, 1H, CH_2_=CH-), 5.62 (dd, *J*_1_ = 1.17 Hz, *J*_2_ = 17.29 Hz, 2H, (*Z*)-CH_2_=CH-), 5.42 (dd, *J*_1_ = 1.17 Hz, *J*_2_ = 10.55 Hz, 2H, (*E*)-CH_2_=CH-), 3.88 (s, 3H, CH_3_-O-), 3.04 (t, *J* = 5.57 Hz, 2H, =N-C-CH_2_-), 2.90 (t, *J* = 5.86 Hz, 2H, -N-C-C-CH_2_-), 2.90-2.85 (m, 1H, CH_2_=CH-CH-), 2.61 (dd, *J*_1_ = 0.88 Hz, *J*_2_ = 3.52 Hz, 2H, Br-CH_2_-CH^*^-), 2.43 (dd, *J*_1_ = 0.88 Hz, *J*_2_ = 6.45 Hz, 2H, Br-CH_2_-CH^*^-), 2.06-1.69 (m, 4H, =C-CH_2_-CH_2_-CH_2_-CH_2_-C=); ^13^C-NMR (chloroform-d_3_): 156.57, 156.14, 153.11, 143.07, 137.04, 130.29, 121.86, 120.91, 120.50, 118.44, 100.91, 55.59, 43.25, 37.58, 33.40, 26.02, 22.96, 22.83; ESI-MS: m/z 361.0 [M]^+^ (calculated for: [C_18_H_22_N_2_O]^+^ 361.3); Elemental analysis: calculated 59.84 % C, 5.86 % H, 7.75 % N; found 60.01 % C, 5.91 % H, 7.65 % N

### 3.4. Molecular docking

Docking calculations were carried out using AutoDockVina 1.1.1. [18]. The structure of *Torpedo californica* AChE (tAChE) was prepared from the crystal structure (pdb code 1acj) using Autodock Tools 1.5.2. [19,20]. The molecular models of ligands were built in ChemDraw 11.0 and minimized with UCSF Chimera 1.3 (Amber Force field) in charged form [21]. Docking calculations were set to 50 runs. A population of 150 individuals and 2,500,000 function evaluations were used. The structure optimization was performed for 27,000 generations using a genetic algorithm. Maximum root mean square tolerance for conformational cluster analysis was 2.0 Å. At the end of calculation, AutoDock Vina performed cluster analysis. The visualisation of enzyme-ligand interactions (Figure 4) was prepared using Pymol 1.1.8. [22].

## 4. Conclusion

In summary, the new cholinesterase inhibitor *N*-(bromobut-3-en-2-yl)-7-methoxy-1,2,3,4-tetrahydroacridin-9-amine (**1**) was synthesized. It was produced by a nucleophilic reaction of 7-MEOTA with (*E*)-1,4-dibromobut-2-ene. This compound was found to be poor inhibitor of cholinesterases *in vitro*, but it showed 15-fold higher selectivity towards hAChE compared to THA. The molecular docking study found different binding of THA or 7-MEOTA compared to compound **1** and explained its low inhibitory ability *in vitro.*

## Figures and Tables

**Table 1:** IC_50_ values of tested compound and standards.

Compound	hAChE IC_50_ ± SD (µM)	hBChE IC_50_ ± SD (µM)	SI hBChE/hAChE
**THA**	0.5 ± 0.1	0.02 ± 0.003	0.05
**7-MEOTA**	15.0 ± 2.4	21.0 ± 3.4	1.4
**1**	89.1 ± 17.3	78.8 ± 12.8	0.88
